# A Multi-Framework Approach to Medication Adherence Evaluation in Pharmacy Student-Led Medication Reviews: An Observational Exploratory Study

**DOI:** 10.3390/pharmacy14030068

**Published:** 2026-04-30

**Authors:** Hanna Keidong, Margit Valge, Kaja-Triin Laisaar, Afonso Miguel das Neves Cavaco, Daisy Volmer

**Affiliations:** 1 Institute of Pharmacy, Faculty of Medicine, University of Tartu, 50411 Tartu, Estonia; margit.valge@ut.ee (M.V.); acavaco@ff.ulisboa.pt (A.M.d.N.C.); daisy.volmer@ut.ee (D.V.); 2 Institute of Family Medicine and Public Health, Faculty of Medicine, University of Tartu, 50411 Tartu, Estonia; kaja-triin.laisaar@ut.ee; 3 iMed.ULisboa, Faculdade de Farmácia, Universidade de Lisboa, 1649-003 Lisboa, Portugal

**Keywords:** medication adherence, education, pharmacy, students, clinical competence, aged, medication review

## Abstract

**Background:** Medication adherence is essential for treatment effectiveness and safety, but pharmacy students may find it difficult to assess adherence comprehensively during medication reviews (MRs). This study examined how pharmacy students assess medication adherence in real-world MRs and explored whether complementary adherence frameworks could support broader evaluation. **Methods:** This observational exploratory study was conducted in the integrated MSc (Master of Science) Pharmacy program at the University of Tartu, Estonia. During the internship, 21 pharmacy students performed a Brown Bag MR with patients aged 65 years or older who used at least 5 prescription medications. Data included patient interviews, e-prescription records, and a validated MR documentation form. An expert panel applied the World Health Organization Medication Adherence Model (WHO-MAM) and the Perceptions and Practicalities Approach (PAPA) to identify adherence determinants not captured by the student-used MR tool. Descriptive statistics and qualitative content analysis were used. **Results:** Students mainly documented therapy- and patient-related issues, such as incorrect dosing, side effects, and interactions, while socioeconomic and healthcare system factors were rarely identified. Students identified potential adherence-related issues in 19% of cases, whereas experts identified such issues in 57% of cases. Additional gaps included limited recognition of financial barriers, access difficulties, and social support factors. **Conclusions:** In this exploratory study, pharmacy students identified medication-use-related problems during MRs, but broader adherence-related determinants were less consistently documented. These preliminary findings suggest that structured frameworks such as WHO-MAM and PAPA may be useful for broadening adherence assessment in experiential pharmacy education.

## 1. Introduction

Chronic diseases often require long-term pharmacotherapy. However, the real-world benefit of treatment depends not only on appropriate prescribing but also on whether patients are able and willing to use medications as intended. Medication nonadherence is common and has been associated with poorer clinical outcomes and higher healthcare costs [1]. Clinically meaningful adherence is not a simple binary construct, as it varies widely depending on the medication, condition, and measurement method [2,3], and is influenced by multiple socioeconomic, therapeutic, and psychological determinants [4]. Importantly, adherence is an integral part of medication use and is closely linked to medication safety [5]. Higher medication literacy of patients improves both adherence and treatment safety [5], whereas adverse effects and complex regimens often compromise adherence and increase the risk of complications [6,7].

The relationship between adherence and health outcomes is not always straightforward, as it may partly reflect broader health-related behaviours. This is illustrated by the healthy adherer effect, in which high adherence even to a placebo has been associated with lower mortality [8]. Adherence should therefore be understood as one component of effective treatment, and improving adherence should be seen as a means of supporting better health outcomes rather than as a separate outcome in itself.

Supporting patients’ medication adherence is an important part of pharmaceutical care and medication safety [9]. To prepare students for this role, pharmacy education should combine theory with practical experience [10], particularly in the context of medication adherence assessment and counselling. Learning about the behavioural and psychosocial determinants behind adherence and practising counselling through role-play [11] helps students connect better with patients. These activities improve communication and empathy and make it easier to notice and address everyday barriers such as forgetfulness, beliefs, or treatment complexity [12].

Pharmacy education on medication adherence varies globally. In Europe, most programs teach the ABC Taxonomy and the clinical impact of nonadherence using diverse teaching methods [13,14]. In the United States, only a few programs offer dedicated courses, and adherence is usually embedded in broader subjects, resulting in limited student familiarity [15]. Educators report insufficient standardized guidance and time constraints [14,15]. In Asia, training focuses on cultural competence, addressing traditional beliefs, language barriers, and family dynamics, with improvements mainly in learners’ cultural understanding [16].

This variation in educational approaches suggests a need for clearer tools to support adherence assessment in practice. Structured frameworks may support a more systematic assessment of adherence-related determinants. Their meaningful application, however, also requires pharmacotherapeutic knowledge and clinical reasoning to interpret medications, treatment goals, adverse effects, regimen complexity, and patient-specific circumstances [17].

In Estonia, this topic is particularly relevant because the national ePrescription system creates opportunities to analyse prescribing and dispensing patterns, including primary medication nonadherence [18] and subsequent dispensing histories, while community pharmacy medication review (MR) services have also been piloted in practice [19,20]. Together, these developments highlight the importance of strengthening pharmacists’ ability to recognize adherence-related problems in routine care, even though the present study focused on adherence assessment during MRs rather than on refill-based adherence measures.

This study examined which adherence-related determinants pharmacy students documented during MRs with geriatric patients experiencing polypharmacy in community pharmacy practice. It further explored to what extent students identified the same adherence-related determinants as an expert panel when classified using the World Health Organization Medication Adherence Model (WHO-MAM) and Perceptions and Practicalities Approach (PAPA). The study also examined which determinants were supported by the student-used MR tool and which were identified only through retrospective expert framework-based classification.

## 2. Materials and Methods

### 2.1. Pharmacy Education in Estonia

The study was conducted within the five-year integrated MSc (Master of Science) Pharmacy program at the University of Tartu, Estonia. The curriculum combines pharmaceutical sciences with clinical pharmacy and patient-centred care. In the 8th semester of studies, the course Practical Social Pharmacy II (5 ECTS) addresses the principles of medication adherence. For example, topics related to different patient groups, their motivation, and education through systematic counselling about medication use are covered. The practical part of the study process is guided through patient–pharmacist role-play cases and immediate feedback from the lecturer. The method also includes a documentation and reflection part, where students fill out their study journals (describing every case, analysing the solution, and offering further insight to improve future counsellings). In the final year (9th–10th semester), students complete a six-month community pharmacy internship, which includes MRs as part of experiential learning [21].

### 2.2. Study Sample

This observational exploratory study used a pragmatic cohort-based sample comprising all pharmacy students who completed the relevant internship assignment during the study period (March–April 2025, *n* = 21). The sample size was determined by the number of eligible student cases available in that academic year rather than by a priori sampling for qualitative saturation. Each student conducted one MR with a geriatric patient (65 years or older, one patient 57 years old*) using at least five prescription medications (over-the-counter medications and supplements were counted separately). The sample was obtained through convenience sampling. Patients with severe cognitive impairment, significant hearing or speech difficulties, terminal illness, or receiving palliative care were excluded. Ethical approval was obtained from the University of Tartu Research Ethics Committee (399/T-2), and written informed consent was obtained from all participants before data collection.

Inclusion criteria: Patients aged 65 years or older who had regularly used at least five prescription medications during the previous two weeks were included. Over-the-counter medications and dietary supplements were also documented where applicable.

Exclusion criteria: Significant hearing or speech impairments, severe cognitive impairment, terminal illness, receipt of palliative care, or admission to an intensive care unit within the previous two months.

* The 57-year-old patient’s data were retained for analysis because the case involved multimorbidity, polypharmacy, a complex medication regimen, and reported side effects.

### 2.3. Medication Review and Data Collection

This assignment represented the students’ first structured clinical interaction with real patients in the context of MR, using a modified Brown Bag approach [22]. Patients were asked to bring all medications used at home, including prescription and over-the-counter products, to the pharmacy for review. These were assessed with the patient using the FIP MR Toolkit structure to identify medication-related and adherence-related issues. The protocol did not include all elements of the original Brown Bag model.

At the first meeting, patients were informed about the purpose of the MR and provided written consent. Supervising pharmacists retrieved prescription and diagnosis data from the national e-prescription database [23], and students entered this information into a MR documentation form adapted from previous Estonian studies, with evidence of face validity [24,25].

**Figure 1:**
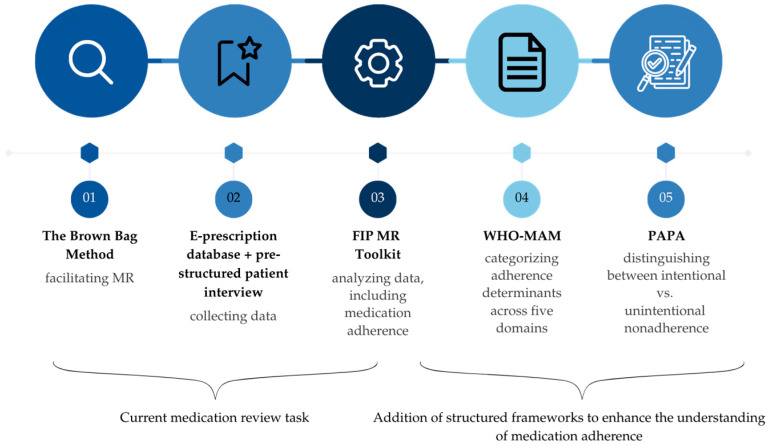
Medication review task components and evaluation (further development) tools.

Secondly, in a patient interview (30–60 min), students assessed medication use, adherence, treatment efficacy, safety, and cost. They documented findings using an MR form and FIP MR Toolkit questions (e.g., ability to follow regimen, barriers, and regimen simplification) [26] (Supplement S1, Figure 1). Students assessed medication adherence using adherence-related questions embedded in the FIP MR Toolkit. No adherence-specific evaluation frameworks were used during the student-led reviews.

The analysis also included a written reflection component, where students engaged in self-reflection on the quality of available information and considered the strengths and limitations of different data sources.

### 2.4. Study Data and Sources

The student-conducted review combined patient interviews with information retrieved from the e-prescription database. The review guide (Supplement S1) was adapted from a form used during the 2019 MR pilot conducted in community pharmacies across Estonia [24]. The MR documentation was altered from an Estonian study published in 2015 [25], which confirmed its relevance and usability for adherence-related purposes. It also demonstrated good face validity, as all participants were able to understand and answer the questions.

Students presented their assessment of medication adherence based on adherence-related questions included in the FIP MR Toolkit [26]. The review addressed four main domains: medication adherence (including the ability to follow the treatment regimen and identification of barriers to adherence); treatment efficacy (covering perceived benefits, unmet health needs, therapeutic dosing, conformity with clinical guidelines, and justification for supplement use); treatment safety (assessing potential or actual side effects, drug interactions, contraindications, and the ongoing appropriateness of therapy); and treatment costs (focusing on the availability of generic alternatives).

Students were prepared for the task through familiarization with the MR documentation form, process, and participation in a seminar covering patient selection procedures and eligibility criteria. Also, pharmacists supervising the internship received corresponding instructions regarding the MR implementation process.

### 2.5. Data Analysis

To explore which medication adherence determinants were captured through the student-used MR tool in real-world settings and to identify patterns in their documentation and reasoning, combined quantitative and qualitative approaches were applied. All personal identifiers were removed before analysis to ensure confidentiality. The study was based on secondary data.

For the purposes of the data analysis, cases were grouped according to the presence or absence of documented adherence-related issues in the available material. Cases in which one or more adherence-related issues were identified were classified as “potential adherence-related issues identified,” while cases with no such issues documented were classified as “no documented adherence-related issues identified.” This grouping was used to support comparison between student documentation and expert framework-based assessment and should be understood as an analytical categorization rather than a definitive assessment of patient adherence.

Adherence determinants were categorized using the following frameworks:

1. FIP MR Toolkit—used by students for the structured medication use and adherence assessment during MR;
2. World Health Organization Medication Adherence Model (WHO-MAM, [27])—used by experts to capture the five domains (socioeconomic, healthcare system-related, condition-, therapy-, and patient-related) of medication adherence determinants;
3. Perceptions and Practicalities Approach (PAPA, [28])—used by experts to distinguish between intentional (belief-driven) and unintentional (practical) determinants of adherence.

WHO-MAM and PAPA were integrated to complement the FIP MR toolkit and provide a multidimensional interpretation of adherence determinants. To support structured comparison, determinants identified by students and the expert panel were mapped into a combined WHO-MAM and PAPA matrix.

WHO-MAM ensures consideration of system-level and socioeconomic determinants often overlooked by students, while PAPA clarifies whether nonadherence stems from beliefs or practical barriers. In this study, the WHO-MAM and PAPA frameworks were applied retrospectively by the expert panel and were not used by the students. Instead, they were used as analytical frameworks to explore additional possibilities for medication adherence evaluation beyond those supported by the MR tool. The expert panel consisted of three experienced practicing pharmacists with teaching experience, selected purposively based on their expertise in pharmacy practice, MRs, and pharmacy education. The panel did not conduct additional interviews with the 21 patients; rather, the analysis was based solely on the student documentation and available case materials.

The panel jointly reviewed the student documentation and conducted a qualitative content analysis through discussion-based consensus. For each case, the experts examined the documented information together, identified adherence-related determinants, and mapped these to the WHO-MAM and PAPA frameworks. Cases in which at least one adherence-related issue was identified were classified as “potential adherence-related issues identified,” whereas cases with no identified adherence-related issues were classified as “no documented adherence-related issues identified” within the available material. This classification was used as an operational category for the exploratory analysis rather than as a definitive binary judgment of patient adherence.

The qualitative coding and framework mapping were performed manually; no qualitative analysis software was used. Two student assignments contained incomplete adherence documentation; however, the remaining components of these submissions (medication use data) were retained in the analysis.

The study is reported in accordance with the STROBE (Strengthening the Reporting of Observational Studies in Epidemiology) Statement, and a completed STROBE checklist is provided as Supplementary Material (Supplement S3).

## 3. Results

### 3.1. Participant Characteristics

A total of 21 pharmacy students completed MRs with 21 geriatric polypharmacy patients, assessing various aspects of medication use, including adherence. The mean patient age was 75 years (range 65–90; one patient was 57 years old), with 16 females and 5 males. On average, patients used 7.8 prescription medications, 1.9 OTC medications, and 3.4 dietary supplements.

### 3.2. Medication-Related Problems Identified by Students

Using the MR tool, adherence documentation predominantly reflected therapy- and patient-related determinants. Students documented a range of medication-related issues, most frequently incorrect administration (frequency, dose, and timing) in 19 cases; experienced side effects in 11 cases; medication interactions in 11 cases; low medication literacy in 8 cases; and independent discontinuation of medication in 4 cases. In several cases, students described determinants relevant to adherence without recognising these as adherence determinants.

### 3.3. Adherence Assessment

Using the FIP MR Toolkit, students’ assessments agreed with the expert panel in 11 of the 19* cases. Specifically, both students and the expert panel identified no documented adherence-related issues in 7 cases and potential adherence-related issues in 4 cases. Patient self-reports (Supplement S2) indicated adherence difficulties in 5 cases (24%), whereas the expert panel identified potential adherence-related issues in 12 cases (57%). Students most frequently documented treatment-related determinants, such as side effects and regimen complexity, as well as patient-related determinants, such as incorrect dosing, while socioeconomic and healthcare system-related determinants were rarely captured using the MR tool.

* Two cases were excluded from this specific comparison due to incomplete adherence documentation, resulting in 19 evaluable cases.

### 3.4. Framework-Based Analysis

Qualitative content analysis of student notes by experts revealed that adherence determinants clustered around WHO-MAM domains of patient-, condition-, and therapy-related determinants. Based on the PAPA approach, intentional adherence determinants (e.g., beliefs about medication necessity) were less frequently described than unintentional determinants (e.g., forgetfulness, regimen complexity). The WHO-MAM × PAPA framework revealed socioeconomic and system-level adherence determinants that were rarely identified by students but captured in expert evaluations, providing a comprehensive overview of determinants (Table 1). 

Rather than quantifying the number of determinants identified, the aim was to explore whether applying the WHO-MAM and PAPA frameworks enabled a more comprehensive and structured identification of factors relevant to medication adherence. The comparison between determinants identified by students and specialists was used to demonstrate the added value of these frameworks. Table 1 summarizes determinants explicitly identified as adherence-related by students and those additionally recognized by the expert panel after retrospective framework-based interpretation. Some information relevant to adherence was present in the student documentation (Supplement S1) without being explicitly interpreted by students as an adherence determinant.

To clarify how the data were interpreted, Table 2 presents illustrative de-identified excerpts from the student documentation together with how the information was documented by the students, the expert panel’s interpretation based on the same material, and the corresponding WHO-MAM and PAPA classification. These examples show that some adherence-related determinants were already present in the student documentation but were not always explicitly recognized or interpreted by students as adherence-related issues. Using the same underlying material, the expert panel then applied the WHO-MAM and PAPA frameworks to identify broader adherence-related determinants, describe them more systematically, and better understand their relevance for medication use. This is important because only determinants that are recognized as adherence-related can be addressed through more targeted patient counselling and support.

The observed discrepancy between student documentation and expert assessment suggests differences in the depth and structure of adherence evaluation, rather than direct student error, because the expert panel retrospectively applied adherence-specific frameworks to the same source material.

### 3.5. Practical Aspects of MRs

Students also reported practical barriers, including difficulties in finding eligible patients, limited time, and a lack of private consultation space. Although the direct impact of these factors on review quality was not formally assessed, they may have affected the depth and consistency of the MRs. In addition, limited access to private consultation space remains a practical constraint in at least some Estonian community pharmacies, which is relevant when interpreting the feasibility of patient-centred MR activities in routine practice. Facilitators included access to medication databases, communication skills, and patients’ adequate medication literacy. Approximately 38% of students noted that prior knowledge and skills acquired in the course of Clinical Pharmacy (5 ECTS) supported their ability to conduct the MR.

## 4. Discussion

This observational exploratory study highlights a persistent challenge in pharmacy education: translating structured approaches to medication adherence assessment into practice. Although students were able to identify medication-related problems, their ability to interpret how these issues relate to patient behaviour, influence adherence, and shape manageable risks appeared more limited [29]. Similar findings have been reported internationally, where pharmacy students often struggle to apply structured adherence assessment approaches in real-world patient evaluation [30]. The present study adds to this literature by showing, in an authentic MR setting, that students more readily recognized medication and patient-related determinants, whereas broader contextual determinants were less consistently documented. This pattern suggests a gap between knowledge acquired during training and its application in practice.

It is also important to stress that MR tools provide structure for assessing medication use, and many are not designed specifically for comprehensive medication adherence evaluation [1]. In educational settings, students may therefore rely on tools that capture adherence only implicitly, making it difficult to systematically address behavioural, socioeconomic, and healthcare system-level determinants of adherence [27]. Identifying complementary adherence evaluation frameworks may help strengthen both educational tasks and clinical reasoning in MRs [28].

Several factors may explain why students overlooked system-level and socioeconomic barriers. First, these determinants are less visible during routine MRs and may require proactive questioning about sensitive topics such as cost or access, which students may feel uncomfortable addressing. Fear of offending patients and/or overstepping professional boundaries may further inhibit discussion of these topics [31]. Second, the educational emphasis on pharmacotherapy and safety may lead students to prioritize clinical issues over behavioural and contextual determinants. Similar patterns have been reported globally, where adherence training is often fragmented or embedded within broader subjects, limiting structured exposure to psychosocial determinants [14,15]. Limited clinical experience may reduce students’ confidence in navigating complex conversations [32]. The limited recognition of broader adherence determinants may partly reflect the fact that the students were novice practitioners and were conducting one of their first structured patient-facing clinical tasks.

The variability in students’ documentation also reflects the absence of standardized tools for assessing adherence determinants. While the FIP MR Toolkit provides a basic structure, it does not explicitly guide differentiation between intentional and unintentional nonadherence or ensure consideration of system-level influences. Integrating complementary frameworks such as the WHO Medication Adherence Model (WHO-MAM) [27] and the Perceptions and Practicalities Approach (PAPA) [28,33] can address these gaps. WHO-MAM promotes a multidimensional perspective by including socioeconomic and healthcare system domains, while PAPA distinguishes between belief-driven (intentional) and practical (unintentional) barriers. Applying these frameworks in this study revealed that students focused primarily on patient- and therapy-related determinants, whereas experts identified additional determinants related to cost, access, and social support. This reinforces the value of combining these models to enhance both educational and clinical practice.

Our findings have important implications for the University of Tartu’s pharmacy curriculum design. Structured adherence training should go beyond theoretical lectures and incorporate experiential learning supported by comprehensive frameworks. Simulated patient encounters, role-play [34], and Objective Structured Clinical Examinations (OSCEs) [35] can help students practice identifying diverse determinants and develop patient-centred strategies. Embedding WHO-MAM and PAPA into pharmacy curricula alongside the FIP MR toolkit would provide a more systematic framework for assessing medication adherence. Incorporating an OSCE adherence-counselling station with a structured checklist to distinguish intentional from unintentional nonadherence would further strengthen students’ clinical reasoning and communication skills. Global evidence suggests that such interventions accelerate competence development and improve patient communication competencies, ultimately supporting better health outcomes [35,36].

Comparisons of student assessments, patient self-reports, and expert evaluations revealed inconsistencies, echoing international research that self-report measures often overestimate adherence and fail to capture complex behavioural patterns [37,38]. This underscores the need for triangulation and structured frameworks in both education and practice.

The operational classification of cases according to whether potential adherence-related issues were identified should be interpreted with caution. Medication adherence is not a dichotomous construct, and this approach does not capture variation in the severity, frequency, or clinical relevance of adherence-related behaviours. Therefore, the classification should be understood as an exploratory categorization based on the available documentation rather than as a definitive assessment of patient adherence.

This study focused on how adherence-related issues were identified and documented during MRs and did not incorporate dispensing-based quantitative adherence measures such as MPR, PDC, or refill interval analysis. As a result, the assessment relied on interview-based and documentation-based information, which may not fully capture actual medication implementation or persistence. Future studies could strengthen adherence assessment by combining review-based and framework-based evaluation with objective refill-derived indicators, such as dispensing histories from the Estonian electronic health system, to provide a more comprehensive view of medication use.

### Limitations

This study included a small patient sample (*n* = 21), which limits the generalizability of the findings. This was because each participating student conducted one MR, and the total number of patients, therefore, corresponded to the course enrolment for that academic year. The sample size was limited to one internship cohort and was not based on formal information power or data saturation principles for qualitative research. Accordingly, the findings should be interpreted as exploratory and descriptive. However, the primary purpose was to conduct an observational exploratory study that allowed flexible evaluation of students’ understanding of adherence and approaches used in MR teaching tasks. As students did not apply adherence-specific frameworks themselves, the study does not assess students’ ability to select or use different adherence evaluation tools. Instead, it explores the potential added value of such tools through expert analysis. The results provide a valuable understanding of how these tools could function in practice and form a basis for developing an improved method with a stronger emphasis on medication adherence.

The sample was obtained through convenience sampling, as students selected suitable patients themselves during their pharmacy internship. Patients unable to fully participate in the interview (due to cognitive impairment, hearing, or speech impairments) were excluded for practical reasons. Consequently, individuals with more complex adherence challenges may not have been represented. This could also have created a selection bias, as almost 75% of the participants were female. Nevertheless, patients with both optimal and insufficient medication adherence were represented in the sample. Such a sampling approach enables assessment of how students apply MR principles in realistic settings and offers useful input for improving the adherence-focused components of training and development of the assignment.

One included case did not meet the predefined age criterion, reflecting the use of an existing standardized teaching case set rather than a newly constructed sample. While this represents a deviation from the original eligibility criteria, the study was exploratory and based on a small sample, and no formal sensitivity analysis was performed. The potential effect of this case on the overall findings should therefore be interpreted with caution.

Future studies could adopt more graded or numerical approaches to adherence assessment, including validated adherence measures, to provide a more nuanced understanding of adherence-related behaviours and their clinical significance.

The depth and quality of student-collected data varied considerably. The strong emphasis on patient-related determinants may indicate that students were not leading the process. These variations highlight the importance of structured counselling tools and targeted training to ensure systematic and comprehensive adherence assessment provided by students.

## 5. Conclusions

This exploratory study suggests that pharmacy students may not consistently identify the full range of adherence-related determinants when using a general MR tool. Retrospective application of adherence-specific frameworks by an expert panel identified additional determinants that were not systematically documented in the student reviews. Although these findings should be interpreted cautiously, given the small, pragmatically selected sample and the cohort-based student and patient selection, they suggest that structured frameworks such as PAPA and WHO-MAM may be useful educational supports for broadening adherence assessment in experiential pharmacy training.

## Figures and Tables

**Table 1:** Medication use determinants representing both facilitators of and barriers to adherence.

WHO-MAM Dimension	PAPA: Perceptions (Necessity Beliefs, Concerns, Attitudes) Intentional Processes		PAPA: Practicalities (Barriers, Facilitators) Unintentional Processes	
	Adherence Determinants Described by Students	Additional Determinants Noted by Experts	Adherence Determinants Described by Students	**Additional** **Determinants** **Noted by Experts**
Socioeconomic determinants	-	-	-	Financial difficulties Supporting family member/relative Irregular prescription purchasing
Healthcare system-related determinants	-	Trust in healthcare providers	Off-label prescribing Long-term treatment prescribed when short-term treatment is appropriate	Difficulty obtaining prescriptions Doctor refuses medication adjustment (after experiencing major side effects) Need for dose adjustments based on patient characteristics and condition
Condition- related determinants	Perceived seriousness of the condition	Knowledge of progressive chronic disease course (and possible complications) Disease-related anxiety and fear of exacerbation (asthma, anxiety attacks)	Condition-related functional limitations (vertigo, joint pain, shortness of breath) Fluctuating or episodic symptoms	Lifestyle-disease interaction (COPD and smoking, gluten-free diet) Asymptomatic or weakly symptomatic conditions Multimorbidity
Treatment- related determinants	Concerns about side effects Medication effect experienced too strong or too weak	Perceived polypharmacy burden Preference for original over generic medications Belief that lifestyle supports treatment	Side effects Need for regimen simplification Medication interactions Duplicate medications with the same mechanism Unnecessary medication in the regimen	No side effects/ accustomed to side effects
Patient-related determinants	Fear of dependence Low medication literacy	Understanding of the diagnosis Thankfulness toward healthcare specialists Fear of withdrawal symptoms	Incorrect administration (frequency, dose, timing) Patient discontinued medication independently Medication expiration Absence of counselling Forgetfulness	Medication purchased but not used Medication dispenser/reminder system Developed a habit of regular use

**Table 2:** Illustrative de-identified excerpts from student documentation and expert framework-based interpretation of adherence-related determinants.

Case	De-Identified Student Excerpt	Medication Use Information Documentation by the Student	Expert Framework-Based Adherence Interpretation	WHO-MAM Dimension	PAPA Domain
A	“Patient lives with his son, who makes sure the medications are taken on time and writes instructions on every new package.”	Family support was documented but not explicitly identified as an adherence-related determinant.	Reliance on caregiver support appears to facilitate regular medication use and reduce practical barriers.	Socioeconomic determinant	Practicalities
B	“Patient feels unpleasant constipation from a pain medication and therefore practically does not use it.”	Side effects and reduced use were documented.	Adverse effects appear to discourage medication use and may represent an intentional adherence issue linked to a negative treatment experience.	Treatment-related determinant	Practicalities
C	“The patient has stopped using some medications on her own because she believes she does not need them.”	Independent discontinuation of treatment was documented.	This suggests intentional non-use driven by beliefs about treatment necessity.	Patient-related determinant	Perceptions
D	“Some medications are not used continuously because the prescription is difficult to obtain.”	Difficulty obtaining prescriptions was documented.	Difficulty obtaining repeat prescriptions may create a healthcare system-related barrier to continuous medication use.	Healthcare system-related determinant	Practicalities
E	“The patient has to split two tablets and says the tablet halves often ‘fly away’.”	A practical administration problem was documented.	The dosage form itself creates a handling barrier that may compromise accurate dosing and implementation.	Treatment-related determinant	Practicalities
F	“Sometimes the patient forgets the evening metoprolol dose because she is watching television or doing something else.”	Forgetfulness was documented as a practical medication-use issue.	This reflects a routine-related implementation problem in everyday medication taking.	Patient-related determinant	Practicalities
G	“The doctor stopped prescribing metformin while the patient was in a care home, and blood glucose was not monitored.”	Treatment history and a care gap were documented.	This suggests a healthcare system-related gap in continuity of care that may have influenced long-term medication use and self-management.	Healthcare system-related determinant	Practicalities
H	“The patient feels that too many medications are prescribed for daily use and that this is not justified.”	Concern about the number of medications was documented.	This suggests negative beliefs about treatment burden and necessity, which may reduce motivation to use medications as prescribed.	Patient-related determinant	Perceptions
I	“The patient has anxiety about exacerbations and uses a short-acting bronchodilator relatively often. Fear of COPD worsening also leads the patient to take a tablet of Validol before physical activity.”	Disease-related anxiety and medication use were documented.	Fear of disease exacerbation appears to influence medication-taking behaviour and may shape adherence through condition-related anxiety.	Condition-related determinant	Perceptions

## Data Availability

The datasets presented in this article are not readily available because access is restricted in accordance with the approved study protocol, participant confidentiality requirements, and the data retention plan.

## Supplementary Materials

The following supporting information can be downloaded at: https://www.mdpi.com/article/10.3390/pharmacy14030068/s1, Supplement S1. Medication review analysis task (based on the FIP Medication Review Toolkit). Supplement S2. Medication adherence self-assessment questionnaire. Supplement S3. STROBE Statement.

## Abbreviations

The following abbreviations are used in this manuscript:

MR	Medication review
MSc	Master of Science
OSCE	Objective Structured Clinical Examination
OTC	Over-the-counter (medication)
PAPA	Perceptions and Practicalities Approach
WHO	World Health Organization
WHO-MAM	World Health Organization Medication Adherence Model
