# Supplementary material for: A Multi-Framework Approach to Medication Adherence Evaluation in Pharmacy Student-Led Medication Reviews: An Observational Exploratory Study
Source: Pharmacy (Basel). 2026 Apr 30;14(3):68. doi: 10.3390/pharmacy14030068 (PMC13214646; doi:10.3390/pharmacy14030068)
Supplement: Supplementary file 1 [file pharmacy-14-00068-s001.zip › Supplement S3_Keidong_revised.pdf]

Supplement S3. STROBE Statement - Completed checklist for the manuscript "**A multi-framework approach to medication adherence evaluation in pharmacy student–led medication reviews: an observational exploratory study**".

	Item No.	Recommendation	Page No.	Relevant text from manuscript
**Title and abstract**	1	(*a*) Indicate the study's design with a commonly used term in the title or the abstract	1	A multi-framework approach to medication adherence evaluation in pharmacy student-led medication reviews: an observational exploratory study
		(*b*) Provide in the abstract an informative and balanced summary of what was done and what was found	1	The methods and results sections of the abstract.
**Introduction**				
Background/ rationale	2	Explain the scientific background and rationale for the investigation being reported	2–3	Introduction explains the importance of adherence and the educational gap.
Objectives	3	State specific objectives, including any prespecified hypotheses	3	This study examined which adherence-related determinants pharmacy students documented during MRs with geriatric patients experiencing polypharmacy in community pharmacy practice. It further explored to what extent students identified the same adherence-related determinants as an expert panel when classified using the WHO-MAM and PAPA. The study also

				examined which determinants were supported by the student-used MR tool and which were identified only through retrospective expert framework-based classification.
**Methods**				
Study design	4	Present key elements of study design early in the paper	3-7	Presented in the medication review and data collection section.
Setting	5	Describe the setting, locations, and relevant dates, including periods of recruitment, exposure, follow-up, and data collection	3-7	This observational exploratory study used a pragmatic cohort-based sample comprising all pharmacy students who completed the relevant internship assignment during the study period (03/2025-04/2025, n = 21). Medication reviews were conducted in Estonian retail pharmacies.
Participants	6	(*a*) *Cross-sectional study*—Give the eligibility criteria, and the sources and methods of selection of participants	4	**Inclusion criteria:** patients aged 65 years or older who had regularly used at least five prescription medications during the previous two weeks. Over-the-counter medications and dietary supplements were also documented where applicable.<br>**Exclusion criteria:** significant hearing or speech impairments, severe cognitive impairment, terminal illness, receipt of palliative care, or admission to an intensive care unit within the previous two months.

				The sample was obtained through convenience sampling (each 5th year student conducted one MR).
Variables	7	Clearly define all outcomes, exposures, predictors, potential confounders, and effect modifiers. Give diagnostic criteria, if applicable	5-6	Main outcomes were adherence determinants identified through student MRs and expert application of WHO-MAM and PAPA.<br><br>For the purposes of the data analysis, cases were grouped according to the presence or absence of documented adherence-related issues in the available material. Cases in which one or more adherence-related issues were identified were classified as "potential adherence-related issues identified," while cases with no such issues documented were classified as "no documented adherence-related issues identified." This grouping was used to support comparison between student documentation and expert framework-based assessment and should be understood as an analytical categorisation rather than a definitive assessment of patient adherence.<br><br>The exposure was the student-led MR process using interviews and e-prescription data.<br><br>No confounders or effect modifiers were formally analysed due to the descriptive design.<br><br>Nonadherence classification followed FIP Toolkit questions and expert framework-based assessment.

Data sources/ measurement	8*	For each variable of interest, give sources of data and details of methods of assessment (measurement). Describe comparability of assessment methods if there is more than one group	5	The student-conducted review combined patient interviews with information retrieved from the e-prescription database. The review guide (Supplement 1) was adapted from a form used during the 2019 MR pilot conducted in community pharmacies across Estonia. The MR documentation was altered from an Estonian study published in 2015, which confirmed its relevance and usability for adherence-related purposes. It also demonstrated good face validity, as all participants were able to understand and answer the questions.<br><br>Students presented their assessment of medication adherence based on adherence-related questions included in the FIP MR Toolkit (Figure 1).
Bias	9	Describe any efforts to address potential sources of bias	5	Bias was addressed through use of a standardised MR form and FIP Toolkit questions, triangulation of interview data with e-prescription records, and independent expert panel review with consensus procedures.
Study size	10	Explain how the study size was arrived at	3	Determined by the number of students enrolled in the course (n=21).

Quantitative variables	11	Explain how quantitative variables were handled in the analyses. If applicable, describe which groupings were chosen and why	5	The analysis relied on descriptive statistics. The number and proportion of cases with potential adherence-related issues identified or no documented adherence-related issues identified,

				as well as the presence of specific adherence determinants, were reported.
Statistical methods	12	(*a*) Describe all statistical methods, including those used to control for confounding	6-7	The study used descriptive and qualitative analytical methods. Adherence status was summarised as counts and proportions, and determinants were analysed through qualitative content analysis and mapping to WHO-MAM and PAPA domains.<br><br>No statistical modelling or confounder adjustment was performed, as the study was exploratory and not designed to test associations.
		(*b*) Describe any methods used to examine subgroups and interactions		No analyses of subgroups or statistical interactions were conducted.
		(*c*) Explain how missing data were addressed	6	Two student assignments had incomplete adherence documentation; however, the remaining components of these submissions (medication use data) were included in the analysis.
		(*d*) *Cross-sectional study*—If applicable, describe analytical methods taking account of sampling strategy	3	The sample was obtained through convenience sampling within the pharmacy internship cohort.
		(*e*) Describe any sensitivity analyses		No sensitivity analyses were performed.

**Results**				
Participants	13*	(a) Report numbers of individuals at each stage of study—eg numbers potentially eligible, examined for eligibility, confirmed eligible, included in the study, completing follow-up, and analysed	7	A total of 21 pharmacy students completed MRs with 21 geriatric polypharmacy patients. The mean patient age was 75 years (range 65–90, one patient was 57 years old*), with 16 females and 5 males.<br><br>*The 57-year-old patient's data were retained for analysis because the case involved multimorbidity, polypharmacy, a complex medication regimen, and reported side effects. This case did not meet the predefined age criterion, reflecting the use of an existing standardized teaching case set rather than a newly constructed sample. Although this represents a deviation from the original eligibility criteria, the study was exploratory and based on a small sample, and no formal sensitivity analysis was performed. Therefore, the potential effect of retaining this case on the overall findings should be interpreted with caution.
		(b) Give reasons for non-participation at each stage		N/A
		(c) Consider use of a flow diagram		No flow diagram was included.
Descriptive data	14*	(a) Give characteristics of study participants (eg demographic, clinical, social) and information on exposures and potential confounders	7	A total of 21 pharmacy students completed MRs with 21 geriatric polypharmacy patients, assessing various aspects of medication use, including adherence. The mean patient age was 75 years (range 65–90, one patient was 57 years old), with 16 females and 5

				males. On average, patients used 7.8 prescription medications, 1.9 OTC medications, and 3.4 dietary supplements.
		(b) Indicate number of participants with missing data for each variable of interest	6	Two student assignments contained incomplete adherence documentation; however, the remaining components of these submissions (medication use data) were retained in the analysis.<br><br>No other variables had missing data.
Outcome data	15*	*Cross-sectional study*—Report numbers of outcome events or summary measures	7	Using the FIP MR Toolkit, students' assessments agreed with the expert panel in 11 of 19* cases. Specifically, both students and the expert panel identified no documented adherence-related issues in 7 cases and potential adherence-related issues in 4 cases. Patient self-reports (Supplement 2) indicated adherence difficulties in 5 cases (24%), whereas the expert panel identified potential adherence-related issues in 12 cases (57%). Students most frequently documented treatment-related determinants, such as side effects and regimen complexity, as well as patient-related determinants, such as incorrect dosing, while socioeconomic and healthcare system-related determinants were rarely captured using the MR tool.<br>*Two cases were excluded from this specific comparison due to incomplete adherence documentation, resulting in 19 evaluable cases.

Main results	16	(*a*) Give unadjusted estimates and, if applicable, confounder-adjusted estimates and their precision (eg, 95% confidence interval). Make clear which confounders were adjusted for and why they were included		Only unadjusted estimates were reported. Outcomes were expressed as counts and proportions of nonadherence and frequencies of adherence determinants. The study did not aim to examine associations between exposures and outcomes; therefore, no confounders were identified or adjusted for, and no confidence intervals were calculated.
		(*b*) Report category boundaries when continuous variables were categorized		N/A – the study reported counts and proportions only, no continuous variables were categorised.
		(*c*) If relevant, consider translating estimates of relative risk into absolute risk for a meaningful time period		N/A – no relative risk estimates were generated.

Other analyses	17	Report other analyses done—eg analyses of subgroups and interactions, and sensitivity analyses	8	Additional analyses included qualitative content analysis of student documentation by an expert panel and mapping of adherence determinants using the combined WHO-MAM × PAPA framework to compare student-identified determinants with those identified by experts.<br><br>Qualitative content analysis of student notes by experts revealed that adherence determinants clustered around WHO-MAM domains of patient-, condition-, and therapy-related determinants. Based on the PAPA approach, intentional

				adherence determinants were less frequently described than unintentional determinants.
**Discussion**				
Key results	18	Summarise key results with reference to study objectives	7-11	In line with the study objectives, the results showed that pharmacy students primarily identified therapy- and patient-related medication-related issues, while broader socioeconomic and healthcare system-related adherence determinants were less frequently documented. Compared with the expert framework-based assessment, students identified fewer cases with potential adherence-related issues, suggesting gaps in adherence recognition during student-led medication reviews.
Limitations	19	Discuss limitations of the study, taking into account sources of potential bias or imprecision. Discuss both direction and magnitude of any potential bias	13-14	The sample was obtained through convenience sampling, as students selected suitable patients themselves during their pharmacy internship. Patients unable to fully participate in the interview (due to cognitive impairment, hearing, or speech impairments) were excluded for practical reasons. Consequently, individuals with more complex adherence challenges may not have been represented. This could also have created a selection bias, as almost 75% of the participants were female. Nevertheless, patients with both optimal and insufficient medication adherence were represented in the sample. Such a sampling approach enables assessment of how students apply

				MR principles in realistic settings and offers useful input for improving the adherence-focused components of training and development of the assignment.<br><br>One included case did not meet the predefined age criterion, reflecting the use of an existing standardized teaching case set rather than a newly constructed sample. While this represents a deviation from the original eligibility criteria, the study was exploratory and based on a small sample, and no formal sensitivity analysis was performed. The potential effect of this case on the overall findings should therefore be interpreted with caution.<br><br>Future studies could adopt more graded or numerical approaches to adherence assessment, including validated adherence measures, to provide a more nuanced understanding of adherence-related behaviours and their clinical significance.<br><br>The depth and quality of student-collected data varied considerably. The strong emphasis on patient-related determinants may indicate that students were not leading the process. These variations highlight the importance of structured counselling tools and targeted training to ensure systematic and comprehensive adherence assessment provided by students.
Interpretation	20	Give a cautious overall interpretation of results considering objectives, limitations, multiplicity of	14	This exploratory study suggests that pharmacy students may not consistently identify the full range of adherence-related determinants when using a general MR tool. Retrospective

		analyses, results from similar studies, and other relevant evidence		application of adherence-specific frameworks by an expert panel identified additional determinants that were not systematically documented in the student reviews. Although these findings should be interpreted cautiously given the small, pragmatically selected sample and the cohort-based student and patient selection, they suggest that structured frameworks such as PAPA and WHO-MAM may be useful educational supports for broadening adherence assessment in experiential pharmacy training.
Generalisability	21	Discuss the generalisability (external validity) of the study results	14	This study included a small patient sample (n = 21), which limits the generalisability of the findings. This was because each participating student conducted one MR, and the total number of patients, therefore, corresponded to the course enrolment for that academic year. The sample size was limited to one internship cohort and was not based on formal information power or data saturation principles for qualitative research. Accordingly, the findings should be interpreted as exploratory and descriptive. However, the primary purpose was to conduct an observational exploratory study that allowed flexible evaluation of students' understanding of adherence and approaches used in MR teaching tasks. As students did not apply adherence-specific frameworks themselves, the study does not assess students' ability to select or use different adherence evaluation tools. Instead, it explores the potential added value of such tools through expert analysis. The results provide valuable understanding into how these tools could function in practice

			and form a basis for developing an improved method with a stronger emphasis on medication adherence.
**Other information**			
Funding	22	Give the source of funding and the role of the funders for the present study and, if applicable, for the original study on which the present article is based	N/A (no funding)

*Give information separately for cases and controls in case-control studies and, if applicable, for exposed and unexposed groups in cohort and cross-sectional studies.
