# Supplementary material for: A Multi-Framework Approach to Medication Adherence Evaluation in Pharmacy Student-Led Medication Reviews: An Observational Exploratory Study
Source: Pharmacy (Basel). 2026 Apr 30;14(3):68. doi: 10.3390/pharmacy14030068 (PMC13214646; doi:10.3390/pharmacy14030068)
Supplement: Supplementary file 1 [file pharmacy-14-00068-s001.zip › Supplement S1_Keidong_revised.pdf]

Supplement S1. Medication review analysis task (based on the FIP Medication Review Toolkit).

Medication Review Analysis (MRA, medication review) conducted by the student

The task is to carry out a medication review analysis for an elderly patient who has been using at least five different prescription and over-the-counter medications on a regular basis during the last two weeks, in addition to dietary supplements and natural products, if the patient uses them. The student identifies the patient in cooperation with the on-site practice supervisor.

The following patients are not included in the medication review analysis: patients under 65 years of age; patients over 65 who regularly use fewer than 5 medications; patients aged 65 and older who have hearing or speech difficulties, serious memory impairment, terminal illness, or who are receiving palliative care; and patients who have been treated in an intensive care unit within the last two months.

The medication review analysis is carried out as follows:

At the first meeting, after the patient has purchased their medication from the pharmacy, they are invited to participate in the medication review analysis and the aims of the analysis are explained to them. If the patient agrees to participate, they sign the information and consent form. The form is also signed by the student conducting the analysis and the person coordinating the analysis, for example the practice supervisor. The signed document is stored in a locked cabinet in the pharmacy until the end of the pharmacy practice training, after which it is submitted to the general practice supervisor at the pharmacy practice training summary seminar. A follow-up visit is then arranged. Before the second visit, the practice supervisor prepares an extract from the digital prescription centre, including the patient's prescription medications and diagnoses, and the student enters this information into the documentation form without any personally identifiable data being included, using a separate Excel file.

At the second meeting, the patient comes with all of their medications, dietary supplements, and natural products, and the student conducts an interview with them. The interview is carried out in private conditions, using the counselling room or counselling area with seating available in the pharmacy. The interview lasts approximately 30 to 60 minutes. During the interview, the following information is collected: lifestyle and risk analysis related to medication use; medication adherence; other medications in use that are not recorded in the digital prescription centre; dietary supplements and natural products; and the patient's actual use of medications. No counselling is provided to the patient during the interview.

Based on the collected data, the student performs the medication review analysis using summaries of product characteristics, databases on drug interactions and adverse effects, clinical guidelines, and other information sources. The student submits the completed documentation form together with the analysis based on the collected data to the drug

interaction and adverse effects module. The analysis is reviewed and assessed by the on-site supervisor and a University of Tartu lecturer. If the patient wishes, the student, together with the on-site practice supervisor, provides the patient with a summary of the analysis.

Analysis (interview) questions:

Medication Adherence

- Is the patient capable of using their medications according to the treatment regimen?
- What prevents the patient from using the medication as prescribed?
- Is it possible to simplify the treatment regimen?

Treatment Efficacy

- Does the patient perceive the effect of the medication and/or understand its necessity?
- Does the patient have complaints for which no appropriate treatment has been prescribed (including consideration of non-pharmacological options)?
- Are the doses of all medications within the appropriate therapeutic range for this patient?
- Do current clinical guidelines recommend the use of the given medication?
- Is the use of all dietary supplements justified?

Treatment Safety

- Has the patient experienced any side effects from medications?
- Have any drug-drug or drug-supplement interactions occurred?
- Does the patient have any diseases or conditions that contraindicate the use of certain medications?
- Are all medications still indicated?
- Are there non-pharmacological methods that could be used in the patient's treatment?

Treatment Cost

- Are there more affordable alternatives available for the medications included in the treatment regimen?
