# Supplementary material for: A Multi-Framework Approach to Medication Adherence Evaluation in Pharmacy Student-Led Medication Reviews: An Observational Exploratory Study
Source: Pharmacy (Basel). 2026 Apr 30;14(3):68. doi: 10.3390/pharmacy14030068 (PMC13214646; doi:10.3390/pharmacy14030068)
Supplement: Supplementary file 1 [file pharmacy-14-00068-s001.zip › Supplement S2_Keidong_revised.pdf]

Supplement S2. Medication adherence self-assessment questionnaire.

Please ask the patient to evaluate their health status and medication use on a 5-point scale.

	Very poor	Poor	Satisfactory	Good	Very good
My health is	[ ]	[ ]	[ ]	[ ]	[ ]

	Disagree	Rather disagree	Neutral	Rather agree	Agree
I know enough about my illness(es)	[ ]	[ ]	[ ]	[ ]	[ ]
I understand for what purpose I use my medications	[ ]	[ ]	[ ]	[ ]	[ ]
I am often worried or disturbed about using medications	[ ]	[ ]	[ ]	[ ]	[ ]
Without medications I would be very ill	[ ]	[ ]	[ ]	[ ]	[ ]
I do not understand how my medications improve my health	[ ]	[ ]	[ ]	[ ]	[ ]

Please describe your medication use during the last two weeks:

	Never	Rarely	Sometimes	Often	Always
I forget to take my medication at the correct time	[ ]	[ ]	[ ]	[ ]	[ ]
It is difficult for me to obtain medications from the package	[ ]	[ ]	[ ]	[ ]	[ ]

I mix up different medications	[ ]	[ ]	[ ]	[ ]	[ ]
I lose my medications	[ ]	[ ]	[ ]	[ ]	[ ]
I have difficulty swallowing tablets	[ ]	[ ]	[ ]	[ ]	[ ]
I find it hard to remember medication dosing	[ ]	[ ]	[ ]	[ ]	[ ]
I lack money to pay for medications	[ ]	[ ]	[ ]	[ ]	[ ]
I have experienced side effects from medications	[ ]	[ ]	[ ]	[ ]	[ ]
I am worried about long-term consequences of using medications	[ ]	[ ]	[ ]	[ ]	[ ]
Using medications has caused me other problems	[ ]	[ ]	[ ]	[ ]	[ ]
I use some medications differently from how prescribed	[ ]	[ ]	[ ]	[ ]	[ ]
I have left some medications unused for the above reasons	[ ]	[ ]	[ ]	[ ]	[ ]

Comments (for example, which medications?):

____
